# Help-seeking behaviour in women diagnosed with gynaecological cancer: a systematic review

**DOI:** 10.3399/BJGP.2022.0071

**Published:** 2022-11-15

**Authors:** Pauline Williams, Marie-Claire Rebeiz, Leila Hojeij, Stephen J McCall

**Affiliations:** Aberdeen Centre for Women’s Health Research, Institute of Applied Health Sciences, University of Aberdeen, Aberdeen, UK.; Centre for Research on Population and Health, American University of Beirut, Lebanon.; Centre for Research on Population and Health, American University of Beirut, Lebanon.; Aberdeen Centre for Women’s Health Research, Institute of Applied Health Sciences, University of Aberdeen, Aberdeen, UK; assistant professor, Centre for Research on Population and Health, Department of Epidemiology and Population Health, Faculty of Health Sciences, American University of Beirut, Beirut, Lebanon.

**Keywords:** delays in care, early detection of cancer, general practice, gynaecological cancer, help-seeking behaviour, systematic review

## Abstract

**Background:**

Identifying what prompts or hinders women’s help-seeking behaviour is essential to ensure timely diagnosis and management of gynaecological cancers.

**Aim:**

To understand the factors that influence the help- seeking behaviour of women diagnosed with gynaecological cancer.

**Design and setting:**

Systematic review and narrative synthesis of studies from high-income settings worldwide.

**Method:**

Five databases were searched for studies, of any design, that presented factors related to the help-seeking behaviour of women diagnosed with a gynaecological cancer. Data from the articles were extracted and presented using narrative synthesis, which was both inductive and deductive. The COM-B (capability, opportunity, motivation, behaviour) model of behaviour change was used as a framework.

**Results:**

In total, 21 studies were included in the review. Inductive synthesis presented three main themes of factors related to the help-seeking behaviour of women diagnosed with gynaecological cancer: patient factors, such as knowledge of symptoms; emotional factors, including previous healthcare experience, embarrassment, and trust; and practical factors, including time and resources. Deductive synthesis demonstrated that capability (namely, symptom knowledge), opportunity (having the required time and overcoming the cultural taboos surrounding gynaecological symptoms), and motivation (believing that seeking help is beneficial) are all required to initiate help-seeking behaviour.

**Conclusion:**

Although it is a journey of defined steps, the help- seeking behaviour of women with symptoms diagnosed with gynaecological cancer is influenced by personal and societal factors. Interventions to improve help seeking will need to address the specific identified factors, as well as capability, opportunity, and motivation.

## INTRODUCTION

Almost one in eight cases of cancer affecting women in the UK will be one of the five gynaecological cancers — namely, endometrial, cervical, ovarian, vulval, or vaginal cancer.^1^ Despite UK survival rates improving — and more than doubling in some cases — cancer survival in the UK lags behind that of other European countries.^2^ It has been suggested that differences in survival are due to late-stage presentation as a result of patient delay.^3^

Any diagnostic journey begins with a patient seeking help from a health professional. It is known that embarrassment, fear of cancer, and poor symptom knowledge may affect how quickly patients would present should they develop symptoms of a gynaecological cancer,^4^^–^^6^ but there has been limited exploration of patient-related delays in women who have been diagnosed with a gynaecological cancer.^7^^–^^9^

The importance of cultural issues in detecting cancer early has been highlighted by the James Lind Alliance (a research priority setting partnership between patients, carers, and clinicians).^10^ Such issues surround the intimate nature of gynaecological cancer symptoms, and the investigation and examination necessary to diagnose gynaecological cancers; however, it is not yet known to what degree these cultural issues influence patient help-seeking behaviour and potential diagnostic delay.

**Figure 1.**
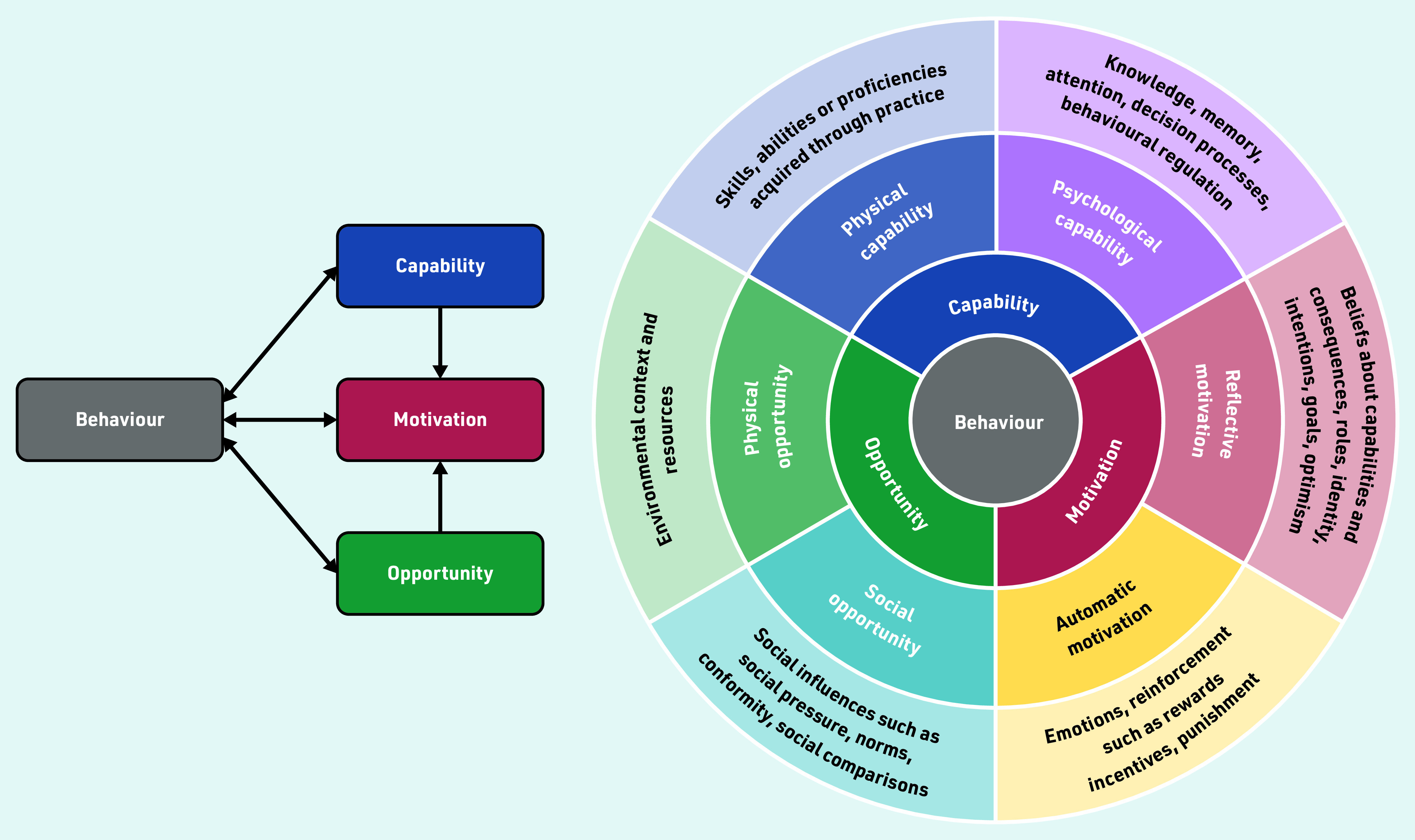
*The COM-B model.^11^© 2011 Michie S, et al ; licensee BioMed Central Ltd. Reproduced with permission and under the terms of the Creative Commons Attribution License (http://creativecommons.org/licenses/by/2.0).*

Using the COM-B (capability, opportunity, motivation, behaviour) behaviour change model (Figure 1)^11^ to identify what prompts, or hinders, patients’ help-seeking behaviour when they have symptoms of gynaecological cancers has the potential to identify targets for intervention that aim to achieve more-timely help seeking. This model suggests that behaviour consists of three components:

- capability — the knowledge and skills needed to engage in help-seeking behaviour;
- opportunity — the external factors that influence help seeking; and
- motivation — the internal processes that influence help seeking.

Identifying the factors associated with patients’ help-seeking behaviour is important and may lead to the development of effective interventions that have the potential to shorten diagnostic delay. This narrative review aimed to understand what factors affect the help-seeking behaviour of women diagnosed with gynaecological cancer.

**Table:** How this fits in

Reducing diagnostic delay by improving patients’ help-seeking behaviour, may reduce the UK’s excess gynaecological cancer mortality. This review identifies that symptom knowledge is not enough to initiate help seeking; patients must also have the time or means to attend health care and be motivated enough, by previous experience, to overcome any fear or embarrassment they may have.

## METHOD

A systematic review was conducted and the protocol was registered on PROSPERO (registration number: CRD42020197677). The Preferred Reporting Items for Systematic reviews and Meta-Analyses (PRISMA) criteria^12^ were followed. Initially performed in June 2020, the search was repeated in March 2022.

### Search strategy

The search strategy (see Supplementary Box S1) included two terms — healthcare seeking and gynaecological cancer — their synonyms, and Medical Subject Headings terms. Searches were conducted in five databases — Cochrane Library, MEDLINE, CINAHL, Embase, and Web of Science — from inception to the time of the study being undertaken. In addition, the reference lists of identified articles were searched manually. The search strategy was developed with input from a medical librarian.

#### Inclusion and exclusion criteria

All original research articles from 1996 until March 2022 were included, be they controlled or uncontrolled quantitative studies, or qualitative studies. Studies were included if they:

- involved patients aged ≥18 years, who had been diagnosed with, or who had symptoms of, a gynaecological cancer;
- involved GPs, trainee GPs, nurse practitioners, gynaecology specialists, and emergency care practitioners; and
- examined the facilitators and barriers to help-seeking behaviour for patients who had been diagnosed with a gynaecological cancer or had symptoms potentially indicative of a gynaecological cancer.

Excluded studies included those that were:

- limited to patients aged <18 years;
- not written in English, or were editorials, unpublished work, or academic theses; and/or
- conducted in medium- and low-income countries, as defined by the World Bank.^13^

#### Study selection

The outputs from the searches were imported into the EndNote reference managing tool, and duplicates were removed. All titles, abstracts, and full- text articles were assessed independently at all stages by two researchers using DistillerSR computer software. All titles were screened against the inclusion and exclusion criteria; abstracts of remaining studies were assessed for eligibility. Any disagreements were resolved by discussion between the researchers. Full texts were obtained for all abstracts that met the inclusion and exclusion criteria.

#### Data extraction and synthesis

Data from the selected full-text articles were extracted independently by two reviewers. Synthesis was narrative and followed the recommended sequence described by Popay *et al*.^14^ Data were analysed using thematic analysis^15^ and framework analysis.^16^ Thematic analysis enabled the emergence of themes and subthemes, while framework analysis was based on the concepts of the COM-B behaviour change model.^11^ Detailed methodology of the data-extraction process is given in Supplementary Box S2.

#### Assessment of data quality

Independent dual quality assessment of each included article was performed using the relevant validated Critical Appraisal Skills Programme (CASP) tool; poor study quality did not affect the inclusion of individual articles.

## RESULTS

**Figure 2.**
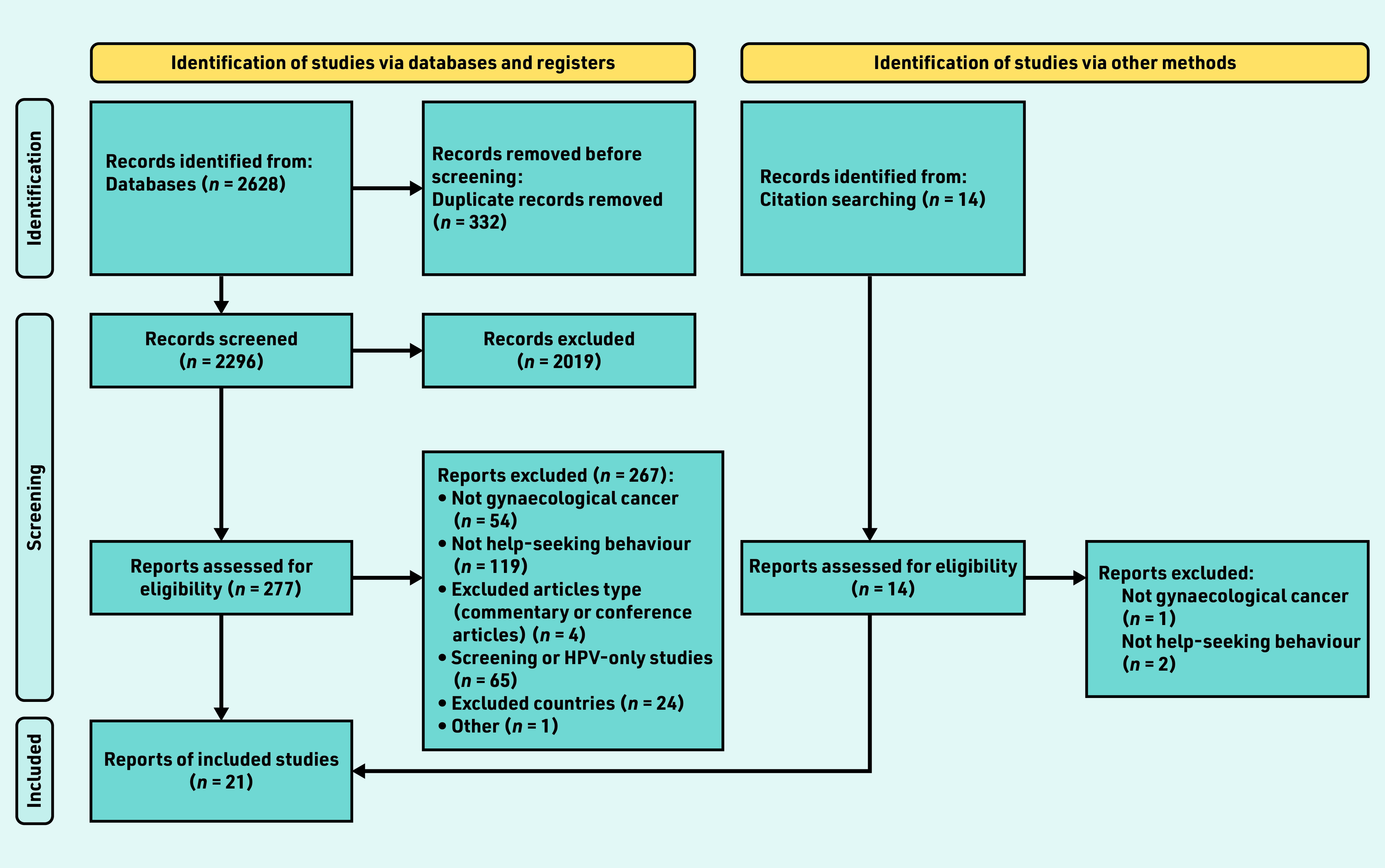
*PRISMA 2020 flow diagram for new systematic reviews that included searches of databases, registers, and other sources. HPV = human papillomavirus.*

A PRISMA diagram outlines the study search and inclusion process (Figure 2). The search identified 2628 titles; in total, 2019 records (titles and abstracts) were excluded during the screening process; of these, 291 full-text articles were assessed for eligibility, with 21 meeting the inclusion criteria.^7^^,^^17^^–^^36^ The reasons for exclusions are shown in Figure 2.

Eight studies were conducted in the UK,^7^^,^^20^^,^^25^^,^^27^^–^^29^^,^^35^^,^^36^ five in the US,^22^^,^^31^^–^^34^ two in New Zealand,^19^^,^^24^ two in Canada,^26^^,^^30^ and one in each of Germany,^18^ Australia,^21^ and Denmark;^17^ one multi-centre study was conducted in both Switzerland and Germany.^23^ Nine studies used qualitative methodology,^18^^–^^21^^,^^23^^,^^25^^,^^26^^,^^30^^,^^36^ seven were cohort studies,^22^^,^^24^^,^^28^^,^^29^^,^^32^^–^^34^ two used mixed methods,^17^^,^^27^ two were systematic reviews,^7^^,^^35^ and one was a cross-sectional study.^31^ A summary of study results can be seen in Supplementary Table S1; quality assessment is shown in Supplementary Tables S2–S4.

**Figure 3.**
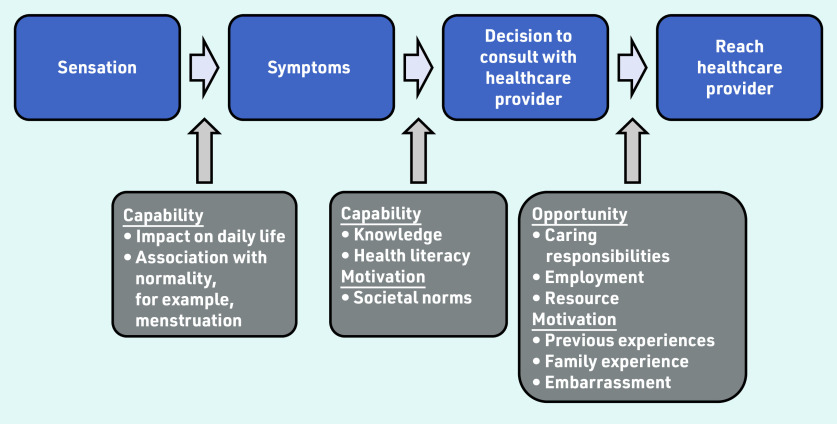
*Factors that influence a patient’s help seeking behaviour*

Individual factors are identified and contextualised to reflect the COM-B behaviour change tool to give an overall assessment of help seeking (Figure 3).

### Main emergent themes

Initial reading of the data identified three themes, and second-level analysis identified several subthemes; these are outlined in Table 1.

**Table 1.** Identified themes and subthemes

**Theme**	**Subtheme**
Patient factors	Symptoms and symptom knowledge
	Interpretation of bodily sensation as symptoms
	Patient demographics

Emotional factors	Fear of finding cancer
	Taboo
	Previous stigmatisation/discrimination
	Legitimisation of symptoms
	Lack of trust in physicians
	Embarrassment
	Previous benign disease

Practical factors	Pressures of time at consultation
	Competing social responsibilities

#### Patient factors

Bodily sensations need to be considered a symptom for patients to seek help; sensations that could be understood in the context of a patient’s life were not determined as symptoms.^17^^–^^20^ When sensations persist, are painful, worsen, interfere with daily life, or have increased permanency, visibility, or palpability, they are more likely to be determined as symptoms.^7^^,^^18^^,^^19^ The years of normal blood loss through menstruation, or even postpartum, lead to some women normalising abnormal vaginal bleeding.^19^^,^^21^^,^^24^

Thirteen articles highlighted the influence of symptom knowledge.^7^^,^^17^^,^^19^^,^^21^^,^^23^^,^^24^^,^^26^^–^^30^^,^^34^^,^^36^ Some women had not heard of the cancer with which they were diagnosed,^19^^,^^23^^,^^30^ while many reported poor symptom knowledge and health literacy.^19^^,^^24^^,^^28^^–^^30^ Symptom misattribution was common, especially in those who had vague symptoms or who were diagnosed with ovarian cancer.^7^^,^^17^^,^^21^^,^^26^^,^^27^^,^^36^

Five studies reported an association between demographic factors and help- seeking behaviour.^17^^,^^24^^,^^28^^,^^29^^,^^31^ Multimorbidity increased delay,^32^ as did being divorced or widowed.^32^ Younger women (aged <25 years) exhibited a delay in help seeking in a study of women diagnosed with cervical cancer,^29^ whereas having a higher socioeconomic status was associated with less delay.^17^ Lawton *et al *^24^ and Ashing-Giwa and Rosales^31^ described the influence of ethnicity on help-seeking behaviour: delay was seen in women of Latin American origin in the US and Maori women in New Zealand.^24^^,^^31^

#### Emotional factors

Fear of finding cancer was found to be an important contributor to delay.^31^^,^^32^^,^^34^ Taught negative attitudes towards gynaecological symptoms was found to contribute to the avoidance of help-seeking behaviour, and such attitudes could cultivate embarrassment, with perceived associations between sexuality and gynaecological symptoms.^19^^,^^23^ Concern about the pain or discomfort associated with the examination could also deter help seeking.^33^ The stigmatisation by clinicians of women who were overweight or the attribution of health complaints to obesity could lead to avoidance of health care.^19^^,^^30^

The influence of family and friends was, however, important, and their encouragement could promote help- seeking behaviour.^18^^,^^20^ The legitimisation of symptoms by clinicians was also found to be important: not explaining about possible causes of symptoms, or not offering alternative diagnoses and advice if symptoms persisted could contribute to patient delay.^35^ A lack of trust in clinicians could also impair help-seeking behaviour,^32^ as could previous benign diagnoses^34^^,^^35^ and the finding that some women feared embarrassment from being considered a hypochondriac or time waster.^20^^,^^34^^,^^35^

#### Practical factors

Caring responsibilities, inconvenient clinic times, and being busy or short of time could all lead to the avoidance of help-seeking behaviour.^19^^,^^20^^,^^33^ The pressure of time required in a consultation to discuss symptoms of what some felt was a private matter also delayed help- seeking behaviour.^19^ In a study of African-American women, having a self-reported barrier to seeking care was associated with prolonged symptom duration prior to presentation, although there was no description of these barriers.^32^ Patients’ prioritisation of others’ health — for example, that of family members — also delayed help-seeking behaviour.^18^^,^^26^

In countries with insurance-based health care, cost could be a deterrent.^33^

### Framework analysis based on the COM-B behaviour change model

#### Capability

Capability refers to whether patients have the knowledge, skills, and abilities required to engage in help seeking. It has two components:

- psychological; and
- physical.

Patients need to know what the symptoms of a disease are; without this, they lack the capability to initiate help seeking.^19^^,^^21^^–^^24^^,^^29^^,^^30^ Many symptoms of gynaecological cancers — especially ovarian cancer — can be vague, and misattribution of these symptoms as non-serious contributed to delays.^7^^,^^17^^,^^25^^–^^27^ Additionally, although vaginal bleeding can indicate an underlying malignancy, it is also a normal physiological process; greater delay was observed in women with vaginal bleeding compared with those with urinary bleeding, which is considered non- physiological.^28^

#### Opportunity

Although capability initiates help-seeking behaviour, women must have the opportunity to access healthcare services in which they have trust. In the context of this model, opportunity refers to the external factors that make help seeking possible; and again, has two components:

- physical — for example, time; and
- social — for example, cultural norms.

Lack of time and being busy could delay help seeking,^19^ as could lack of trust in physicians.^32^ Social responsibilities such as prioritising family members also led to delays.^18^^,^^20^^,^^26^^,^^33^ Opportunity was also determined by social influencers: taboo and embarrassment about the association between gynaecological symptoms and sexuality could lead to delay.^19^^,^^23^ Additionally, not wanting to be seen as a time waster or hypochondriac also contributed to delay.^7^^,^^20^^,^^35^ Discussion with family and friends could reinforce the belief that symptoms are worthy of health care seeking.^18^^,^^20^ Difficulty navigating a healthcare system — for example, because of cost or getting to a clinic or surgery — deterred help-seeking behaviour.^33^

#### Motivation

Motivation refers to the internal processes that influence a patient’s decision to seek help. Its two components are:

- reflective — for example, based on previous experience; and
- automatic — for example, resulting from fear and inhibition.

Fear of finding cancer was an important demotivator to help seeking.^31^^,^^33^^,^^34^ Moreover, previous healthcare experience of stigmatisation and discrimination could reduce motivation, leading to delay.^19^^,^^30^^,^^32^ Examination or symptoms that had previously been reassuring or suggestive of benign disease could decrease motivation,^20^^,^^21^^,^^34^^,^^35^ as could concerns about painful examination.^33^ Renzi *et al* also reported that a lack of explanation for symptoms or lack of advice about what to do if symptoms persisted could also contribute to delay.^35^

## DISCUSSION

### Summary

Help-seeking behaviour is complicated. This narrative review has identified patient, emotional, and practical factors that influence help seeking. Although symptoms and symptom knowledge were highlighted as being key, it is clear that knowledge alone is not sufficient to positively affect help-seeking behaviour. Patients’ previous experiences must motivate them to seek help and the opportunity to do so must be available; health care must be trustworthy, and social responsibilities — such as employment and caring roles — must not impede access.

### Strengths and limitations

This review has been systematically conducted and is the first, of which the authors are aware, to examine the help- seeking behaviour of women diagnosed with a gynaecological cancer using the COM-B framework. The data presented provides a comprehensive summary of the available evidence, as well as highlighting the gaps in knowledge. The combination of thematic and framework analysis has added a robustness to the results.

The studies were mostly observational and their quality varied. They were heterogeneous, cancer types and research methods varied, and outcomes were self- reported and descriptive. There was also heterogeneity in study participants and healthcare systems investigated. As a result, the lack of commonality does not permit definitive conclusions.

### Comparison with existing literature

Patients are aware that early diagnosis is important.^37^ Symptom knowledge is a key element of help-seeking behaviour and others have reported that lack of knowledge can lead to delay,^5^^,^^8^^,^^38^ whereas increased knowledge can positively influence help seeking.^39^^–^^41^ Women’s interpretations of gynaecological cancer symptoms as normal or trivial, or attributing them to pre- existing illness, can lead to delay in help seeking.^20^^,^^40^ Although symptom knowledge has been associated with higher income and higher educational attainment, it has been reported as being lower in older women.^42^

The effect of competing demands has also been described previously,^20^^,^^43^ as has the influence of friends and family.^44^ The positive effect of social support was confirmed by Whitaker *et al*.^40^

The COVID-19 pandemic appeared to alter help-seeking behaviour, with evidence that concerns about overburdening an overstretched healthcare system affected patients’ decision to seek help.^45^ It is unclear whether these changes will persist or change as the course of the pandemic changes.

Worrying about wasting GP time is a known concern for patients and can cause help-seeking delay.^28^^,^^40^^,^^46^ Lack of trust in the healthcare system has also been observed to cause delay.^40^ Fear can both prompt and delay help seeking.^8^^,^^40^^,^^47^

### Implications for practice

Many health-promotion interventions focus on improving knowledge but do not result in improved cancer diagnosis.^48^ Although symptom knowledge is important, women’s decisions to seek help are also influenced by societal norms and previous experiences of help seeking. This review highlights areas of patient behaviour that have potential for intervention, for example, improving symptom knowledge.

Societal demands — for example, care responsibilities — are difficult to influence. Time pressures during consultations and fragmentation of primary care have been reported as contributing to decreased patient centredness, which is associated with reduced symptom reporting;^46^^,^^49^ the Royal College of General Practitioners and Royal College of Obstetricians and Gynaecologists have suggested that primary care consultations be increased to 15 minutes,^50^^,^^51^ but the mismatch between resources and demand makes this a challenging target. When asked what they believed were the biggest barriers to presentation for women with gynaecological cancer symptoms, GPs cited lack of awareness and vagueness of symptoms — that is, capability — suggesting that reluctance to present was best managed by patient education.^52^ Only 14% of GPs questioned felt improving access to health care would reduce presentation delay.^52^

In the same study by Evans *et al*, GPs also cited embarrassment as a barrier to presentation.^52^ Reluctance to discuss health concerns is associated negatively with help seeking.^53^ Although it has been shown that many women are embarrassed to discuss gynaecological health with clinicians, much of the evidence is anecdotal or from third- sector surveys.^54^ More research is needed to explore the influence of embarrassment, and societal and cultural influences, surrounding gynaecological cancer and its symptoms on patient behaviour and help seeking.

Although a journey of defined steps, help-seeking behaviour of women with symptoms who are diagnosed with gynaecological cancer, is influenced by personal and societal factors. Interventions to improve help seeking will need to address the identified factors, as well as capability, opportunity, and motivation.
